# The effect of a maternal meal on fetal liver blood flow

**DOI:** 10.1371/journal.pone.0216176

**Published:** 2019-06-12

**Authors:** Gun Lisbet Opheim, Tore Henriksen, Guttorm Haugen

**Affiliations:** 1 Department of Fetal medicine, Oslo University Hospital—Rikshospitalet, Oslo, Norway; 2 Norwegian Advisory Unit on Women`s Health, Oslo University Hospital—Rikshospitalet, Oslo, Norway; 3 Institute of Clinical medicine, University of Oslo, Oslo, Norway; 4 Department of Obstetrics, Oslo University Hospital—Rikshospitalet, Oslo, Norway; INIA, SPAIN

## Abstract

**Introduction:**

During the third trimester of development, the human fetus accumulates fat, an important energy reservoir during the early postnatal period. The fetal liver, perfused by the nutrient-rich and well-oxygenated blood coming directly from the placenta, is assumed to play a central role in these processes. Earlier studies have linked fetal liver blood flow with maternal nutritional status and response to the maternal oral glucose tolerance test. Our aim was to explore the effect of a regular maternal meal on fetal liver blood flow at two timepoints during the third trimester, representing the start and towards the end of the fetal fat accretion period. We also sought to explore the influence of prepregancy body mass index on how the maternal meal affects fetal liver blood flow.

**Methods:**

Using ultrasound Doppler, we examined 108 healthy women with singleton pregnancies in gestational weeks 30 and 36. At each visit, the first examination was performed with the participant in a fasting state at 08.30 a.m., followed by a standard breakfast meal of approximately 400 kcal. The examination was repeated after 105 minutes. Umbilical vein and ductus venosus blood flow was estimated from diameter and blood flow velocity measurements. Fetal liver flow was calculated as umbilical vein flow minus ductus venosus flow, and change in liver blood flow as flow after minus before the meal. The total group was divided into a normal-weight group (prepregancy body mass index 18.5–25.0 kg/m^2^; n = 83) and an overweight group (prepregancy body mass index >25.0 kg/m^2^; n = 21). Four women with prepregancy body mass index <18.5 kg/m^2^ were excluded from these analyses. Non-parametric statistical hypothesis tests were used for group comparisons.

**Results:**

For the total group, we observed a significant increase in median (10^th^ - 90^th^ percentile) liver flow 28.9 (‒67.9–111.6) ml/min (p = 0.002) following the meal in week 36, but not in week 30, ‒2.63 (‒53.2–65.0) ml/min (p = 0.91). This result in turn yielded a statistically significant increase in delta liver flow from weeks 30 to 36 of 26.0 (‒107.1–146.6) ml/min (p = 0.008). The increase in postprandial liver flow was observed only in the normal-weight group in week 36. Accordingly, the delta liver flow values between the two weight groups were significantly different in week 36 (p = 0.006) but not in week 30 (p = 0.155). Among the normal-weight women, the increase in delta liver blood flow from weeks 30 to 36 was 39.3 (‒83.0–156.1) ml/min (p<0.001); in contrast, we observed no statistically significant change in the overweight group (‒44.5 (‒229.0–123.2) ml/min; p = 0.073). As a substitute for liver size, we divided the delta liver flow values by abdominal circumference and found no changes in the statistical significance results within or between the two weight groups.

**Conclusion:**

In our healthy study population, we observed a statistically significant difference in liver blood flow after maternal intake of a regular meal. This effect depended on gestational age and maternal prepregancy body mass index, but apparently was independent of liver size, based on abdominal circumference as a proxy measure.

## Introduction

Population-based studies have shown that nutritional conditions during fetal life may determine short and long-term health [1, 2]. This association has led to the hypothesis that the fetal environment mediates long-term effects on phenotypic characteristics through epigenetic changes [3, 4]. For example, mortality from coronary heart disease shows a U-shaped relation with neonatal abdominal circumference (AC) [5]. Fetal growth is regulated by genetic disposition and placental transfer of nutrients and oxygen in addition to factors related to maternal body composition and diet [6].

In normal pregnancies, the ability of the fetus to store fat during the third trimester is crucial for meeting the energy demand of the fetal brain during the first postnatal days. The fetal liver is assumed to have an important role in this process through its synthesis of glucose, lipids, and growth factors [7, 8]. It is therefore reasonable that blood flow through the liver is involved in the mechanisms underlying the liver’s regulatory role in fetal energy metabolism. Tchirikov et al., using fetal sheep, reported a six-fold increase in cell proliferation in the liver after obstructing ductus venosus (DV) blood flow and increasing liver blood flow [9]. Under non-hypoxic conditions, both maternal and fetal factors influence blood flow distribution to the fetal liver [10, 11]. One predominant determinant is the balance between the blood pressure gradient across the DV and vascular resistance within the liver [12]. The mechanisms underlying these changes in liver flow are largely unknown but may involve liver vascular structure, endocrine factors, the autonomic nervous system, and local endothelial-derived factors. The relative importance of these factors in adjusting liver flow may vary with the gradual maturation of the liver [13–16].

In the human, fetal fat accretion occurs largely during the third trimester. In this period, maternal food intake and nutritional status may be significant modifiers. Neonatal fat mass is positively related with fetal liver blood flow in gestational weeks 30 and 36 [11]^,^ [17] i.e., representing the beginning and towards the end of the energy-depositing phase. The positive correlation seems to be stronger for mothers with a normal prepregnant body mass index (ppBMI) compared to overweight mothers [17]. Haugen et al. measured fetal liver blood flow in week 31 before and after a maternal glucose tolerance test. They found that flow changes were positively related to AC, with larger fetuses distributing more blood to the liver after a glucose test compared to smaller fetuses [18]. However, a glucose tolerance test represents a non-physiological challenge.

Fetal liver blood flow thus is conceivably related to the accretion of fat during fetal life. It is also reasonable to anticipate that even within the normal physiological range, liver blood flow might show a fine-tuned response to a regular maternal meal, depending on maternal nutritional status, placental function, and fetal size and age.

Our primary aim was to investigate the influence of a standard breakfast meal (SBM) on fetal liver blood flow at two gestational periods in the third trimester in healthy mothers and fetuses. We also examined associations with maternal ppBMI of blood flow distribution to the fetal liver before and after a regular meal.

## Material and methods

In this prospective cohort study, we included 137 healthy women of Caucasian origin with singleton pregnancies. They were approached at the routine ultrasonographic examination at 18–20 weeks of gestation. Exclusion criteria were maternal diseases that affect pregnancy, serious complications during a previous pregnancy, medication with overt potential to affect fetoplacental and fetal circulation, fetal malformations and food intolerance or food allergy. Data were collected from January 2014 to July 2016.

We calculated estimated date of delivery using measures of head circumference (HC) in gestational weeks 18–20 [19]. Doppler ultrasound examinations were performed at 30 and 36 weeks of gestation, and neonatal weight was measured at birth. One clinician (GLO) performed all examinations, each lasting no more than 40 min. The same equipment was used for all participants (Acuson Sequoia 512; Mountain View, CA, USA) with a curved transducer (frequency 2–6 MHz). The fasting examination was performed in the morning (08:30 a.m.). The SBM consisted of two slices of bread with cheese and ham, one boiled egg, one standard package with jam and one with butter, and one glass (about 150 ml) with milk and one glass with juice, for a caloric intake of about 400 kcal. The examination was repeated about 105 min after the meal (standard deviation of 10 and 8 min in weeks 30 and 36, respectively).

Internal vessel diameter (D) and time-average maximum velocity (TAMX) were measured in the straight portion of the intra-abdominal umbilical vein (UV) and at the inlet of DV [20]. The vessel diameters (D_UV_ and D_DV_) were measured and calculated as the mean of five to ten repeated measurements [21]. TAMX was measured during a period of 3–5 s (UV) or as the mean velocity of three consecutive heart cycles (DV). Middle cerebral artery Doppler velocity waveforms were sampled from the proximal part near the circle of Willis [22]. Umbilical artery Doppler traces were sampled in a free-floating loop. All measurements were performed during fetal quiescence [21].

For velocity measurements, insonation angle was kept as low as possible and corrected for. The median angles for all TAMX_UV_ and TAMX_DV_ measures were 8 ° and 16 °, respectively.

The intra-observer variation was analyzed as the intra-class correlation coefficient by means of one-way random-effects ANOVA. The resulting values were 0.99 for D_UV_ (n = 20) and 0.96 for D_DV_ (n = 21).

UV blood flow (Q_UV_) was calculated as 0.5·(D_UV_/2)^2^·π·TAMX_UV_ and DV blood flow (Q_DV_) as 0.7·(D_DV_/2)^2^·π·TAMX_DV_, where 0.5 and 0.7 represent the coefficients for the spatial blood velocity profile in the UV and DV, respectively [23]. Umbilical venous liver blood flow (Q_liver_) was defined as Q_UV_ minus Q_DV_ and the ratio of UV blood shunted through DV (DV_ratio_) as Q_DV_/Q_UV_. Change in liver blood flow (ΔQ_liver_) was calculated as Q_liver_ after minus before the meal.

Fetal biometric measurements included AC, HC, and femur length. We applied z-scores of fetal biometric measurements using Norwegian growth curves [19] and birthweight [24]. The birthweight z-scores were adjusted for gestational age at birth and for sex.

Pre- and postprandial venous blood samples were collected in sodium heparin vacutainers after each ultrasound examination, kept on ice, and centrifuged within 20 minutes (6°C, 2500 ×*g*, 20 min). The supernatants were removed and stored at -80°C. Glucose was measured by an accredited laboratory (Department of Medical Biochemistry, Oslo University Hospital) using the hexokinase/glucose-6-phosphate dehydrogenase enzymatic in vitro test (Roche, Mannheim, Germany).

### Statistics

Descriptive data are presented as median values with percentiles (10^th^–90^th^) and frequencies with percentages, as appropriate. Because of the skewed distribution of flow parameters before and after SBM and for some of the delta values, the Wilcoxon Signed-Rank test was used to compare the median values between two paired samples. We used the Mann–Whitney U and Kruskal–Wallis tests to analyze group differences in delta values between independent samples. Correlations were determined by Spearman’s rank correlation. All statistical tests were two-sided, and we considered p<0.05 to indicate statistical significance. Data were analyzed using SPSS version 23.0 (SPSS Inc., Chicago, IL, USA).

### Ethical approval

The study was approved by Oslo University Hospital Institutional Review Board, reference number 2013/14124 and the Regional Ethics Committee, REK Sør-Øst 2013/1327 Norway, and followed the standards laid out in the Helsinki Declaration. Participants gave written informed consent. All procedures followed institutional guidelines.

## Results

We had a complete dataset for 108 participants, with measures of Q_UV_ and Q_DV_ before and after the SBM in both gestational weeks 30 and 36. Of the 137 participants originally included, eight were excluded. The reasons for exclusion were severe early growth restriction (n = 1), trisomy 21 (n = 1), malformations (n = 1), information about embryo donation (n = 1), or birth before gestational week 36 (n = 4). The remaining 21 women were excluded because of one or more missing flow measurements. For maternal and neonatal characteristics, see Table 1.

**Table 1:** Maternal and neonatal characteristics. Maternal and neonatal characteristics in the total group of included pregnancies (n = 108), normal-weight maternal group (ppBMI^a^ 18.5–25.0 kg/m^2^, n = 83), and overweight maternal group (ppBMI >25.0 kg/m^2^, n = 21). Four women were defined as underweight (ppBMI<18.5 kg/m^2^) and excluded from the comparisons.

	Total group n = 108	Maternal normal-weight group n = 83	Maternal overweight group n = 21	Mann–Whitney U test
**Maternal characteristics:**	Median (10^th^–90^th^ percentile) n (%)	Median (10^th^–90^th^ percentile) n (%)	Median (10^th^–90^th^ percentile) n (%)	p value
Maternal age (years)	32 (28–40)	32 (28–41)	31 (26–38)	0.356
Prepregnant body mass index (ppBMI)	22.3 (19.2–26.5)	21.8 (19.4–24.2)	27.5 (25.4–30.3)	
Glucose level in fasting state week 30 (mmol/L)	4.7 (4.4–53)	4.7 (4.4–5.2)	4.9 (4.5–5.4)	0.049
Postprandial glucose level week 30 (mmol/L)	5.2 (4.1–6.2)	5.1 (4.1–6.2)	5.5 (4.2–6.5)	0.222
Glucose level in fasting state week 36 (mmol/L)	4.7 (4.3–5.2)	4.6 (4.2–5.2)	4.9 (4.5–5.2)	0.017
Postprandial glucose level week 36 (mmol/L)	5.6 (4.4–7.1)	5.5 (4.3–7.0)	5.9 (4.6–7.3)	0.058
**Fetal and neonatal characteristics:**				
Gestational age at 1st study visit (weeks ^+ days^)	30^+1^ (29^+2^–31^+0^)	30^+1^ (29^+2^–30^+6^)	30^+2^(29^+0^–31^+0^)	0.570
Gestational age at 2nd study visit (weeks ^+ days^)	36^+1^ (35^+1^–36^+6^)	36^+0^ (35^+1^–36^+6^)	36^+2^ (35^+1^–37^+2^)	0.357
Gestational age at delivery (weeks ^+ days^)	40^+1^ (38^+5^–41^+4^)	40^+1^ (38^+4^–41^+4^)	39^+6^ (39^+3^–42^+0^)	0.592
Abdominal circumference (AC), week 30 (mm)	264.4 (252.7–274.0)	263.7 (254.7–273.7)	266.5 (250.7–276.2)	0.400
Abdominal circumference (AC), week 36 (mm)	324.2 (310.3–338.1)	323.7 (309.2–336.6)	328.0 (315.0–339.6)	0.122
Birthweight (g)	3569 (3127–3997)	3535 (3120–3986)	3660 (3260–4228)	0.192
Birthweight z-score	−0.26 (−1.02–0.75)	−0.27 (−1.07–0.77)	−0.08 (−0.68–1.15)	0.267
Placental weight (g)	666 (512–842)	659 (511–811)	720 (470–948)	0.162
Nulliparous	69 (63.9)	55 (67.1)	12 (57.1)	0.438
Males	51 (47.2)	40 (48.2)	8 (38.1)	0.409
**Background Doppler data:**				
Umbilical artery ^a^PI, fasting state week 30	0.95 (0.76–1.15)	0.94 (0.75–1.14)	0.98 (0.73–1.32)	0.308
Umbilical artery PI, fasting state week 36	0.84 (0.65–1.06)	0.83 (0.63–1.07)	0.85 (0.65–1.05)	0.716
Middle cerebral artery PI, fasting state week 30	2.18 (1.72–2.54)	2.17 (1.72–2.55)	2.16 (1.60–2.56)	0.930
Middle cerebral artery PI, fasting state week 36	1.92 (1.45–2.45)	1.91 (1.44–2.51)	1.96 (1.44–2.30)	0.996
Ductus venosus PI, fasting state week 30	0.53 (0.35–0.71)	0.53 (0.34–0.70)	0.52 (0.24–0.68)	0.549
Ductus venosus PI, fasting state week 36	0.46 (0.28–0.70)	0.48 (0.29–0.71)	0.46 (0.26–0.71)	0.192

^a^ PI: pulsatility index

To calculate the volume blood flow in DV and UV, we used the diameter and velocity measurements presented in S1 Table.

**Fig 1:**
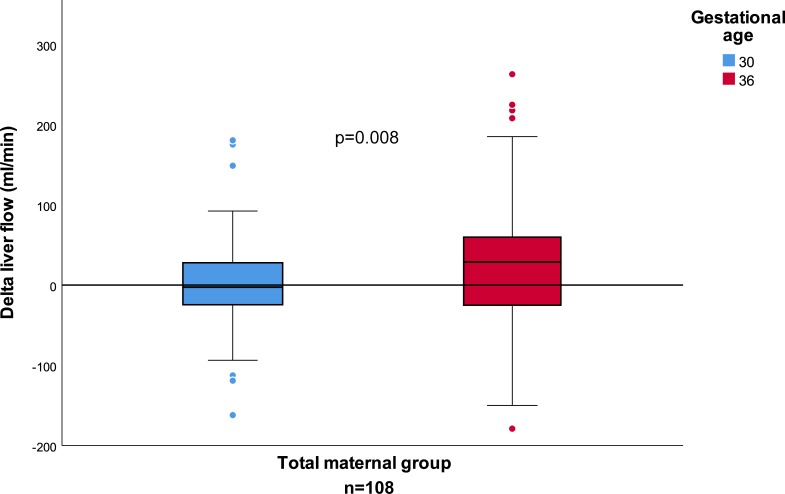
Change in fetal liver blood flow in gestational weeks 30 and 36 for the total maternal group. Box-plot shows the Δliver blood flow at 30 (blue) and 36 (red) weeks of gestation. There was a significant positive change in Δliver flow from weeks 30 to 36.

Among the 108 pregnancies, Q_UV_, Q_DV_, DV_ratio_, and Q_liver_ did not change statistically significant following SBM in week 30. In week 36, Q_UV_ and Q_liver_ increased significantly (p = 0.003 and p = 0.002, respectively) after the meal (Table 2), but DV_ratio_ and Q_DV_ did not. The increase in ΔQ_liver_ from week 30 to 36 was significant (26.0 (‒107.1–146.6) ml/min, p = 0.008) (Fig 1) and remained significant after dividing ΔQ_liver_ by AC (p = 0.011) (Table 3).

**Table 2:** Fetal blood flow measurements for the total group. Blood flow in the umbilical vein, ductus venosus, and fetal liver and the ratio shunted through ductus venosus^a^ during the fasting state and 105 min after a standard breakfast meal for the total group (n = 108) in gestational weeks 30 and 36.

	Fasting state	105 min postprandial	Wilcoxon Signed-Rank test	^b^Δ flow value
**Gestational age 30 weeks**	Median (10^th^−90^th^ percentile)	Median (10^th^−90^th^ percentile)	p value	Median (10^th^−90^th^ percentile)
Umbilical vein flow (ml/min)	149.7 (104.8–236.4)	145.8 (107.7–255.6)	0.706	0.65 (−51.0–68.9)
Ductus venosus flow (ml/min)	38.5 (20.9–61.0)	38.0 (20.1–75.9)	0.120	1.76 (−15.0–22.3)
^a^Ratio, ductus venosus shunting	0.249 (0.131−0.433)	0.249 (0.135−0.478)	0.240	0.003 (−0.10–0.15)
Liver flow (ml/min)	109.4 (67.9–191.1)	108.6 (60.0−197.0)	0.907	−2.63 (−53.2–65.0)
**Gestational age 36 weeks**				
Umbilical vein flow (ml/min)	211.5 (146.0–315.5)	231.5 (161.4–375.5)	0.003	24.11 (−70.1–116.9)
Ductus venosus flow (ml/min)	57.9 (28.3–102.7)	58.3 (27.4–106.3)	0.944	0.96 (−26.5–24.8)
^a^Ratio, ductus venosus shunting	0.251 (0.139−0.489)	0.239 (0.112−0.478)	0.087	−0.02 (−0.18–0.14)
Liver flow (ml/min)	152.8 (78.1–245.1)	176.8 (88.0−245.1)	0.002	28.90 (−67.9–111.6)

^a^ Ratio shunted through ductus venosus: ductus venosus flow divided by umbilical vein flow

^b^ Δ flow value: flow after minus before meal

**Table 3:** Δliver flow measures and changes, divided by abdominal circumference. Δliver flow divided by AC (ml/min/cm) in week 30 and 36, and Δliver flow change/AC from week 30 to week 36, for the total maternal group (n = 108), the normal-weight maternal group (ppBMI^a^ 18.5–25.0, n = 83), and overweight maternal group (ppBMI >25.0, n = 21).

	Δ Liver flow/AC Week 30 (ml/min/cm)	Δ Liver flow/AC Week 36 (ml/min/cm)	Wilcoxon Signed-Rank test	Δ Liver flow Change/AC (ml/min/cm)
Maternal weight group:	Median (10^th^−90^th^ percentile)	Median (10^th^−90^th^ percentile)	p value	Median (10^th^−90^th^ percentile)
Total maternal group (n = 108)	−0.1 (−19.7–24.3)	8.8 (‒ 19.3‒34.4)	0.011	9.1 (−38.1–45.1)
Normal ^a^ppBMI (n = 83)	−1.1 (−23.0–20.9)	10.3 (−13.4–37.0)	<0.001	11.9 (−27.3–51.5)
High ppBMI (n = 21)	−0.9 (−14.9–51.9)	−10.1 (−43.4–31.7)	0.054	−12.3 (−82.6–38.9)
Mann–Whitney U test p value	0.144	0.005		0.002

^a^ ppBMI: prepregnant body mass index

### Correlations

ΔQ_liver_ showed a negative correlation with maternal ppBMI in week 36 (r_s_ = ‒0.21; p = 0.029), but not in week 30 (r_s_ = 0.07; p = 0.47). The ΔQ_liver_ did not correlate with AC z-score in week 30 or 36 or with placental weight or birthweight z-score. We found no associations with sex. We observed a significant positive correlation of fetal birthweight z-score with maternal ppBMI (r_s_ = 0.22; p = 0.025) and placental weight (r_s_ = 0.44; p<0.001), but not between ppBMI and placental weight (r_s_ = 0.10, p = 0.31).

Birthweight z-score also was positively correlated with fasting glucose at week 30 (r_s_ = 0.27; p = 0.006) and with fasting and postprandial glucose at week 36 (r_s_ = 0.32; p = 0.002 for both), but not with postprandial glucose at week 30 (r_s_ = ‒0.07; p = 0.53). Maternal ppBMI was positively correlated with week 30 and week 36 fasting glucose (r_s_ = 0.21; p = 0.032 and r_s_ = 0.26; p = 0.011, respectively), but not with postprandial glucose values (r_s_ = 0.13; p = 0.199 and r_s_ = 0.20; p = 0.066, respectively).

### Maternal ppBMI groups

Of the 108 participants, 83 women had ppBMI between 18.5 and 25.0 kg/m^2^ (normal-weight group) and 21 had ppBMI >25 kg/m^2^ (overweight group) (Table 1). Four women were defined as underweight and were not included in further analyses. Except for a significant difference in maternal fasting glucose levels in both weeks 30 and 36, values for maternal, fetal, or neonatal variables did not differ significantly between the two maternal weight groups.

In the normal-weight group (n = 83), Q_UV_, Q_DV_, DV_ratio_, and Q_liver_ did not change statistically significant following the meal in week 30. In week 36, Q_UV_ and Q_liver_ increased significantly after the meal (p<0.001 for each), and DV_ratio_ decreased (p = 0.013) (Table 4). For the overweight group (n = 21), these flow variables did not differ significantly in week 30 or 36 (Table 5).

**Table 4:** Fetal blood flow measurements for the normal-weight group. Blood flow in the umbilical vein, ductus venosus, and fetal liver and the ratio shunted through the ductus venosus^a^ during the fasting state and 105 min after a standard breakfast meal for the normal-weight maternal group (ppBMI^a^ 18.5–25.0, n = 83) in gestational weeks 30 and 36.

	Fasting state	105 min postprandial	Wilcoxon Signed-Rank test	^b^Δ flow value
**Gestational age 30 weeks**	Median (10^th^−90^th^ percentile)	Median (10^th^−90^th^ percentile)	p value	Median (10^th^−90^th^ percentile)
Umbilical vein flow (ml/min)	148.2 (106.7–236.4)	142.5 (107.8–237.2)	0.775	0.49 (−15.6–63.2)
Ductus venosus flow (ml/min)	38.5 (20.3−60.3)	35.6 (20.3−70.7)	0.126	2.03 (−15.5–23.2)
^a^Ratio, ductus venosus shunting	0.256 (0.136–0.407)	0.251 (0.136−0.474)	0.197	0.003 (−0.09–0.15)
Liver flow (ml/min)	110.3 (70.6–190.2)	108.5 (59.7–188.1)	0.516	−2.84 (−61.7–52.8)
**Gestational age 36 weeks**				
Umbilical vein flow (ml/min)	209.9 (141.2–304.9)	233.2 (162.1–384.6)	<0.001	26.53 (−53.6–124.7)
Ductus venosus flow (ml/min)	55.7 (25.5–100.3)	58.2 (25.3–94.8)	0.817	1.50 (−26.5–25.5)
^a^Ratio, ductus venosus shunting	0.278 (0.135−0.497)	0.221 (0.110−0.441)	0.013	−0.03 (−0.17–0.13)
Liver flow (ml/min)	151.6 (71.9–239.5)	180.0 (99.4–322.1)	<0.001	31.93 (−42.5–124.2)

^a^ Ratio shunted through ductus venosus: ductus venosus flow divided by umbilical vein flow

^b^ Δ flow value: flow after minus before meal

**Table 5:** Fetal blood flow measurements for the overweight group. Blood flow in the umbilical vein, ductus venosus, and fetal liver and the ratio shunted through ductus venosus^a^ during the fasting state and 105 min after a standard breakfast meal for the overweight maternal group (ppBMI^a^ >25.0, n = 21) in gestational weeks 30 and 36.

	Fasting state	105 min postprandial	Wilcoxon Signed-Rank test	^b^Δ flow value
**Gestational age 30 weeks**	Median (10^th^−90^th^ percentile)	Median (10^th^−90^th^ percentile)	p value	Median (10^th^−90^th^ percentile)
Umbilical vein flow (ml/min)	156.0 (90.7–240.9)	154.9 (102.9–316.9)	0.181	11.31 (−30.0–122.1)
Ductus venosus flow (ml/min)	40.4 (21.3–92.6)	45.3 (15.5–97.0)	0.986	−2.33 (−20.3–24.9)
^a^Ratio, ductus venosus shunting	0.31 (0.11–0.58)	0.22 (0.10–0.68)	0.614	−0.004 (−0.23–0.11)
Liver flow (ml/min)	100.1 (47.2–205.6)	115.6 (45.7–271.8)	0.205	−2.41 (−39.8–133.8)
**Gestational age 36 weeks**				
Umbilical vein flow (ml/min)	234.2 (165.4–370.4)	228.1 (132.4–310.9)	0.230	−22.07 (−137.6–97.9)
Ductus venosus flow (ml/min)	61.1 (36.8–106.5)	64.5 (36.8–121.2)	0.455	1.03 (−26.9–57.0)
^a^Ratio, ductus venosus shunting	0.24 (0.13–0.40)	0.32 0.12–0.44)	0.192	0.05 (−0.17–0.27)
Liver flow (ml/min)	171.2 (95.3–241.5)	161.6 (65.6–277.6)	0.217	−32.03 (−145.2–108.3)

^a^ Ratio shunted through ductus venosus: ductus venosus flow divided by umbilical vein flow

^b^ Δ flow value: flow after minus before meal

**Fig 2:**
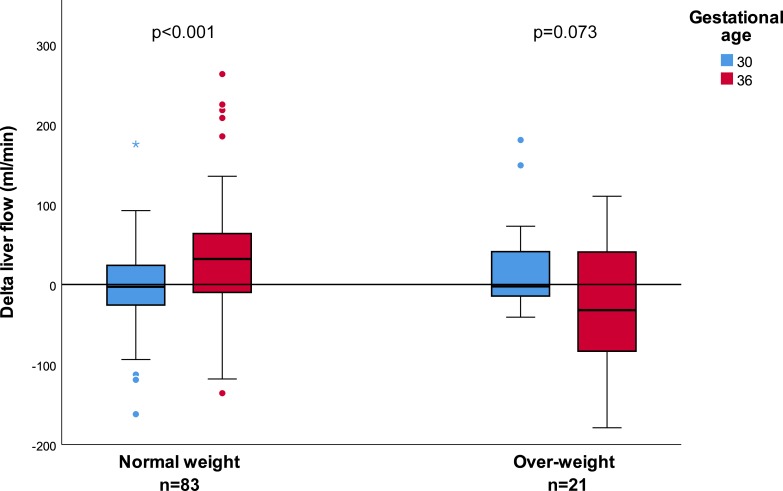
Change in fetal liver blood flow in gestational weeks 30 and 36, between two maternal weight groups. Box-plot shows the Δliver blood flow at 30 (blue) and 36 (red) weeks gestation stratified by maternal weight (median values): the normal-weight group (ppBMI^a^ 18.5–25.0 kg/m^2^, n = 83) and the overweight group (ppBMI >25.0 kg/m^2^, n = 21). In week 36, the Δliver flow was significantly different between the two maternal groups (p = 0.006), but not in week 30 (p = 0.155).

In the normal-weight group, these changes resulted in a significant increase in ΔQ_Liver_ from week 30 to week 36 of 39.3 (‒83.0–156.1) ml/min (p<0.001). In contrast, for the overweight group, ΔQ_Liver_ showed no statistically significant change during this period; ‒44.5 (‒229.0–123.2) ml/min (p = 0.073). ΔQ_Liver_ was statistical significantly different between the two groups in week 36 (p = 0.006) but not in week 30 (p = 0.155) (Fig 2). Contributing to this difference in the later period were a significantly higher ΔQ_UV_ (p = 0.005) and lower ΔDV_ratio_ (p = 0.041) for the normal-weight group compared to the overweight group. Dividing the ΔQ_Liver_ by AC did not change the significance levels within or between the two maternal groups (p = 0.144 in week 30 and p = 0.005 in week 36) (Table 3).

The maternal subgroups did not differ significantly in ductus venosus, umbilical and fetal middle cerebral artery pulsatility indices before food intake in week 30 or 36, Table 1.

## Discussion

In the total group of 108 pregnancies, we observed a significant increase in UV and liver blood flow in week 36 after food intake, but not in week 30. Of note, we identified this effect of the meal only in the normal ppBMI group, with a tendency to the opposite effect among those with high ppBMI. Furthermore, the results showed a significant postprandial increase in liver flow from week 30 to week 36 in the normal-weight group only. In fact, in the high-ppBMI group, postprandial liver flow was decreased between 30 and 36 weeks. This result persisted after adjustment for AC as a measure of fetal size [25].

We thus found a gestational age–dependent effect of a maternal meal on fetal liver flow. The physiological maturation of the fetal liver may play a role in its ability to regulate blood flow following nutrient exposure. Adjustment for AC, as an indicator of general growth, did not change the results, so inherent functional changes could underlie this effect. The mechanisms behind the regulation of fetal liver blood flow are elusive. The presence of a DV “sphincter” is controversial, and more smooth muscle is found in the intrahepatic branches of the portal vein than in the DV isthmus [26]. The degree of DV shunting might be regulated by pressure gradients arising from increased or decreased resistance of the hepatic vascular bed; if so, the suggestion is of an influence of the autonomic nervous system as well as nitric oxide production of the sinusoid endothelial lining [16]. Sympathetic stimulation is believed to aid contraction of the hepatic artery, and parasympathetic stimulation causes sinusoidal relaxation [15]. In the rat, the endothelial cells lining the hepatic sinusoids produced nitric oxide, modulating the flow resistance [27].

We also observed that ppBMI influenced the effect of a maternal meal on fetal liver flow in late gestation. A relationship between fetal liver flow and maternal nutritional status has been reported previously [10, 11]. Ikenoue et al. described fetal liver flow as correlating positively with newborn body fat percentage as early as gestational week 30 [17]. This correlation was stronger for mothers with ppBMI below compared to above 25 kg/m^2^. Haugen et al. found that fetuses of mothers with low fat percentage and/or poor diet had higher liver flow at 36 weeks of gestation. These authors suggested a compensatory increase in liver flow for fetuses with lower nutritional resources to improve energy storage before birth [10].

Comparing the normal-weight and overweight maternal groups, we observed a significantly higher ΔQ_Liver_ in the normal-weight group in week 36 (Fig 2), regardless of fetal size (Table 3). In fact, the positive ΔQ_Liver_ value in the normal-weight group contrasted with the negative value in the overweight group. Of note, the only significant difference between these two maternal groups was fasting glucose level. In human endothelial cell culture, prolonged elevated glucose levels seem to modulate and even reduce the vasodilatory effect of nitric oxides [28]. In overweight mothers, the normal fetal liver response to nutrition might be impaired because of their “over-nourished” environment. Supporting this idea is the recent observation of lipid accumulation in liver in the offspring of obese compared to normal-weight baboons [14]. Those authors concluded that maternal obesity alters pathways central to lipid and glucose metabolism.

In contrast, fetuses of mothers in the normal-weight group might have been more sensitive to regular maternal nutritional intake and regulated liver flow response depending on their predisposed growth potential. A significant increase in UV flow in addition to a significant reduction in DV shunting after food intake contributed to increased liver flow in week 36. This association indicates both placental and fetal responses, involving liver flow after altered hepatic vascular responses as part of a liver maturation process [13]. Thus, in the last part of pregnancy, when fetal need for nutritional resources increases, systemic and local mechanisms regulating the circulatory adjustments among the placenta, fetus, and fetal liver might depend on the trajectory of early maternal nutritional supply within the normal physiological range.

Roland et al. showed that placental weight, parity, maternal weight gain, and body mass index are independent determinants of fetal growth and birthweight [29]. In the current study, we found no correlation between the delta liver flow values in week 30 or 36 and corresponding AC z-scores or birthweight z-score. This result contrasts with the findings of our previous study on fetal liver flow following 75 g maternal glucose loading [18]. The discrepancy might be explained by the different interventions, with a larger impact of glucose load compared to a balanced nutritional meal.

The placenta may influence the effect of maternal nutrition on fetal growth by two pathways. First, it may set the capacity for transplacental transport, dependent on a direct gradient (e.g., glucose). Second, placental interference with the nutrient supply to the fetus may depend on the nutritional needs of the placenta itself.[30] We speculate that for overweight mothers, the first pathway dominates, giving fetuses a disproportionately rich nutrient supply out of the homeostatic range for fetal liver blood flow. These fetuses may be subjected to increased risk for future obesity and insulin resistance/type 2 diabetes [31]. The second pathway may operate more often in the normal-weight population, resulting in an interchange between placenta and fetus. In cases of insufficient nutrition for the fetoplacental unit, the fetus may develop a “thrifty phenotype” with increased risk for non-communicable diseases later in life [32, 33].

We examined the fetuses in weeks 30 and 36, representing the beginning and near the end of the energy-depositing period of fetal growth. A low intra-observer variation supports good reliability of the measurements. The study is limited by the lack of a comparison group unexposed to food. We thus cannot exclude that the observed liver flow changes are part of cyclic variations. However, preliminary results in a similar study from our group showed no altered fetal liver flow during continuous fasting in term pregnancies. Additionally, cyclic variations conceivably also could be so regular in time and space that they could explain our observations. Compared to the non-breathing status, fetal respiratory movements increase both UV diameter and blood flow velocities [34], and for this reason, we performed our measurements in fetal quiescence. However, an influence of fetal movements on our results cannot be excluded. We did not measure hepatic arterial and portal vein blood flow. Portal vein blood flow is not considered a source of nutrient substrates in the fetus because of minimal nutritional uptake from the gut [35, 36].

## Conclusion

In our healthy study population, we found significant differences in liver flow after food intake, dependent on gestational age and maternal ppBMI, but apparently independent of AC, as a substitute for liver size. These fetal adjustments might affect postnatal life, and the results emphasize the importance of maternal nutritional status for the fetus, with an acknowledged influence on future health.

## Supporting information

S1 Table Diameter and velocity measurements of the umbilical vein and ductus venosus. Measurements used for calculation of volume blood flow in the umbilical vein and ductus venosus.
(DOCX)

S1 Dataset Raw data file for the present study. (ZIP)
