# Supplementary material for: The effect of a maternal meal on fetal liver blood flow
Source: PLoS One. 2019 Jun 12;14(6):e0216176. doi: 10.1371/journal.pone.0216176 (PMC6561550; doi:10.1371/journal.pone.0216176)
Supplement: S1 Table — Measurements used for calculation of volume blood flow in the umbilical vein and ductus venosus. (DOCX) [file pone.0216176.s001.docx]

**S1 Table. Diameter and velocity measurements of the umbilical vein and ductus venosus.**

Measurements used for calculation of volume blood flow in the umbilical vein and ductus venosus.

|  | Fasting state | 105 min postprandial |
| --- | --- | --- |
| **Gestational age 30 weeks** | Median  (10^th^ – 90^th^ percentile) | Median  (10^th^ – 90^th^ percentile) |
| Umbilical vein diameter (mm) | 5.63  (4.85 – 6.33) | 5.58  (4.77 – 6.20) |
| Umbilical vein time-average maximum velocity (cm/s) | 21.1  (16.0 – 29.3) | 21.8  (15.8 – 27.7) |
| Ductus venosus diameter (mm) | 1.56  (1.11 – 1.99) | 1.56  (1.12 – 1.97) |
| Ductus venosus time-average maximum velocity (cm/s) | 52.4  (38.9 – 63.3) | 51.9  (38.5 – 64.6) |
|  |  |  |
| **Gestational age 36 weeks** |  |  |
|  |  |  |
|  |  |  |
| Umbilical vein diameter (mm) | 6.67  (5.72 – 7.66) | 6.66  (5.68 – 7.70) |
| Umbilical vein time-average maximum velocity (cm/s) | 21.5  (13.4 – 29.1) | 23.2  (15.8 – 32.2) |
| Ductus venosus diameter (mm) | 1.83  (1.41 – 2.37) | 1.84  (1.37 – 2.38) |
| Ductus venosus time-average maximum velocity (cm/s) | 51.0  (36.8 – 64.5) | 53.1  (38.2 – 64.5) |
